# Participation of obstetric nurses in the care of labor and delivery in private hospitals in Brazil: a cross-sectional observational study

**DOI:** 10.1590/1806-9282.20260119

**Published:** 2026-08-07

**Authors:** Alessandra Costa Timoteo, Maria Cristina Gabrielloni, Rosemeire Sartori de Albuquerque, Edward Araujo, Marina Macruz Rugna, Gustavo Yano Callado, Sue Yazaki Sun, Márcia Barbieri

**Affiliations:** 1 Universidade Federal de São Paulo, Escola Paulista de Enfermagem, Sector of Woman Health – São Paulo (SP), Brazil.; 2 Universidade de São Paulo, School of Arts, Sciences, and Humanities – São Paulo (SP), Brazil.; 3 Universidade Federal de São Paulo, Escola Paulista de Medicina, Department of Obstetrics – São Paulo (SP), Brazil.; 4 Universidade Municipal de São Caetano do Sul, Discipline of Woman Health – São Caetano do Sul (SP), Brazil.; 5 Faculdade Israelita de Ciências da Saúde Albert Einstein, Discipline of Woman Health – São Paulo (SP), Brazil.

**Keywords:** Labor, Delivery, Obstetric nursing, Cesarean section

## Abstract

**OBJECTIVE:**

The aim of this study was to evaluate the participation of obstetric nurses in care provided during labor and delivery within the Brazilian private health system.

**METHODS:**

This cross-sectional observational study included women admitted for delivery in six private hospitals—five in São Paulo and one in Santos, Brazil. Data were obtained through structured interviews conducted during the postpartum period and included information on hospital admission, labor and delivery care, healthcare professionals involved, and obstetric interventions. Women with live births during the study period were included.

**RESULTS:**

A total of 2,435 postpartum women were interviewed. Among them, 48.2% were in their first pregnancy, and 56.4% were primiparous. Only 14.9% had a previous vaginal delivery, while 31.2% had undergone a previous cesarean section. Labor was attended by a physician in 99.3% of cases. Although 52.3% of women had the presence of an obstetric nurse during labor, only 0.7% received delivery assistance from this professional. Among women with labor onset, 58.3% had a vaginal delivery, and only 3.2% were assisted exclusively by obstetric nurses during labor and delivery. Overall, 76.6% of participants underwent cesarean section.

**CONCLUSION:**

Obstetric nurses were frequently present during labor but rarely performed delivery care in private hospitals. Their limited participation suggests underutilization of this professional group within the studied setting.

## INTRODUCTION

Approximately 2.6 million births occur annually in Brazil, involving nearly six million individuals, with 98% taking place in healthcare facilities across public and private sectors^1^. In the private healthcare sector, cesarean section rates reach 85.5%, substantially exceeding the levels recommended by the World Health Organization for the country (25–30%)^2^. This pattern raises important concerns regarding the quality, safety, and appropriateness of obstetric care.

Efforts to improve maternal and neonatal outcomes are currently guided by the Sustainable Development Goals, which emphasize the reduction of preventable maternal and neonatal morbidity and mortality, as well as the promotion of respectful, evidence-based care^3^. In Brazil, national policies such as the Rede Cegonha (Stork Network), implemented in 2011, have reinforced the importance of humanized childbirth and recognized obstetric nurses as key professionals in the care of low-risk pregnancies^4^. Despite these advances, challenges persist in translating recommendations into routine clinical practice, particularly in the private sector, where medicalized models of care remain predominant^5^.

Globally, the State of the World’s Midwifery Report (2021), developed by the United Nations Population Fund in collaboration with the World Health Organization and the International Confederation of Midwives, highlights a shortage of approximately 1.1 million professionals in sexual, reproductive, maternal, neonatal, and adolescent health, including around 900,000 midwives^6^. These professionals are capable of delivering up to 90% of essential care and play a central role in improving outcomes when adequately integrated into health systems^7^.

Evidence indicates that obstetric nurses provide high-quality, evidence-based, and woman-centered care, with the potential to reduce unnecessary interventions and improve maternal and neonatal outcomes^6,8^. In Brazil, expanding their participation could contribute to addressing the excessive use of cesarean sections and promoting best practices in both public and private healthcare settings^9^. However, the extent to which these professionals are effectively integrated into care—particularly within the supplementary health sector—remains insufficiently documented.

In this context, there is a clear gap in knowledge regarding the actual participation of obstetric nurses in labor and delivery care in private hospitals in Brazil. Understanding their role in this setting is essential to inform policies and strategies aimed at improving the quality of obstetric care. Therefore, this study aimed to evaluate the participation of obstetric nurses in labor and delivery care within the Brazilian supplementary health system, using a cross-sectional observational design, and to provide data relevant for improving care models in the private sector.

## METHODS

### Study design and data source

This is a cross-sectional observational study based on secondary data from the “Healthy Birth: Prospective evaluation of the implementation and effects of a multifaceted intervention to improve the quality of childbirth and birth care in hospitals in Brazil,” coordinated by the Oswaldo Cruz Foundation. The original study included 42 health institutions selected according to delivery volume, cesarean section rates, geographic macro-region, urban or rural location, and proportion of private hospital beds.

### Study setting and selection of hospitals

For the present analysis, six private hospitals participating in the Adequate Birth Project were included, five located in São Paulo and one in Santos. This subset was selected to evaluate obstetric care within private institutions engaged in a structured quality improvement initiative. Although this approach allows for a focused analysis, it may limit the generalizability of the findings to other settings.

### Participants

The study included all women admitted for delivery during the data collection period and their newborns, including live births of any gestational age or birth weight and stillbirths at ≥20 weeks of gestation and/or ≥500 g.

### Data collection procedures

Data were collected between March and August 2017 using two standardized electronic instruments. The first consisted of structured interviews conducted with postpartum women at least six hours after delivery, in private settings, including weekends and holidays. This instrument collected sociodemographic, obstetric, prenatal, and labor and delivery information, as well as maternal perceptions of care.

The second instrument involved abstraction of maternal and neonatal data from medical records, including admission characteristics, care during labor and delivery, healthcare professionals involved, and interventions performed.

Interviewers underwent prior training and standardization according to the original study protocol, and data were collected electronically to minimize transcription errors. Information obtained from interviews and medical records was cross-checked when applicable to improve data consistency.

### Sampling strategy

Approximately 400 women were recruited per hospital using randomized daily lists, ensuring representation of births across different times of day and days of the week. Due to the use of secondary data, detailed information on refusal rates and losses was limited but considered minimal in the original study.

### Statistical analysis

Descriptive analyses were performed to characterize the study population and the participation of obstetric nurses in labor and delivery care. Categorical variables are presented as absolute and relative frequencies, and numerical variables as means, medians, ranges, and standard deviations, as appropriate.

Given the descriptive nature of the study, no inferential statistical tests or adjustments for clustering were performed. Analyses were conducted using Stata 19. Missing data were handled by complete-case analysis, and the number of observations for each variable is reported when applicable.

### Ethical considerations

The study was approved by the Ethics Committee of the Federal University of São Paulo (protocol 1.761.057; CAAE 50147415.3.0000.5240). Authorization to use the dataset was granted by the Healthy Birth study coordination, and informed consent was obtained from all participants in the original study.

## RESULTS

A total of 2,435 postpartum women were interviewed in six private hospitals within Brazil’s supplementary health system, located in São Paulo and Santos, using secondary data from the Healthy Birth study.

As shown in Table 1, participants had a relatively homogeneous sociodemographic profile: 34.5% were aged 30–34 years, 63.6% identified as white, 47.5% had 11–14 years of schooling, and 91.7% were married or living with a partner. Most (77.4%) reported paid employment, and 98.0% had private health insurance.

**Table 1:** Sociodemographic characteristics of women in the puerperium period.

Variable	n	%
Age (years)	2,435	100.0%
10–19	58	2.4%
20–24	236	9.7%
25–29	504	20.7%
30–34	840	34.5%
35–39	635	26.1%
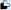 40	161	6.6%
Without information	1	0.0%
Race		
White	1,550	63.6%
Black/Mixed	819	33.7%
Asian	62	2.5%
Indigenous	2	0.1%
Without information	2	0.1%
Education level		
1–10 years of schooling	127	5.2%
11–14 years of schooling	1,156	47.5%
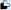 15 years without postgraduate degree	634	26.0%
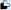 15 years with postgraduate degree	509	21.0%
Without information	9	0.3%
Marital status		
Single	167	6.8%
Married/living with the partner	2,230	91.7%
Separated/divorced	29	1.2%
Widow	1	0.0%
Without information	8	0.3%
Paid employment		
No	543	22.3%
Yes	1,884	77.4%
Without information	8	0.3%
Health insurance		
No	55	2.3%
Yes	2,379	97.7%
Without information	1	0.0%

Nearly half of the women were in their first pregnancy (48.2%), and 56.4% were primiparous. Previous vaginal delivery was reported by 14.9%, while 31.2% had a prior cesarean section. Among the 1,061 women with previous deliveries, 760 (71.6%) had a cesarean section and 62 (5.8%) had both vaginal and cesarean deliveries (Table 2).

**Table 2:** Obstetrical history of women in the puerperium period.

Variable	n	%
Number of pregnancies, including the actual	2,435	100
1	1,174	48.2%
2	769	31.6%
3	320	13.1%
4	122	5.0%
5	36	1.5%
6–10	13	0.5%
Without information	1	0.0%
Number of previous miscarriages	458	100%
1	362	79.0%
2	74	16.2%
3–5	21	4.6%
Without information	1	0.2%
Number of deliveries	2,435	100
Primiparous	1,373	56.4%
1–2	1,001	41.1%
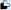 3	60	2.5%
Without information	1	0.0%
Previous cesarean section	760	100
Yes	760	
Without information	2	0.1%
Mean±SD	1.2±0.5	
Median (minimum–maximum)	1.0 (1.0–5.0)	
n	760	
Previous vaginal delivery	363	100
Yes	363	
Without information	2	0.1%
Mean±SD	1.3±0.6	
Median (minimum–maximum)	1.0 (1.0–6.0)	
n	363	
Type of pregnancy	2,435	100
Singleton	2,355	96.7%
Twin	80	3.3%

SD: standard deviation.

At hospital admission, 76.7% had not experienced premature rupture of membranes and 59.8% had not entered labor. Labor was attended by a physician in 99.3% of cases, who had also provided prenatal care for 53.6% of participants. Although 52.3% of women had an obstetric nurse present during labor, only 0.7% received delivery assistance directly from this professional.

Among women who experienced labor onset, 58.3% had a vaginal delivery. Only 3.2% were assisted exclusively by obstetric nurses during labor and delivery. Overall, 76.6% of participants underwent cesarean section; among these, 55.6% were primiparous and 72.1% had not gone into labor prior to surgery (Table 3).

**Table 3:** Characteristics and the healthcare professional responsible for assisting the labor.

Variable	n	%
Premature rupture of the ovular membranes at admission	2,435	100
No	1,866	76.7%
Yes, clear fluid	438	18.0%
Yes, fluid with meconium	40	1.6%
Yes, fluid with blood	3	0.1%
Yes, purulent fluid	1	0.0%
Yes, unspecified	84	3.5%
Without information	3	0.1%
Cervical dilatation (cm)		
Mean±SD	1.8±2.3	
Median (minimum–maximum)	1.0 (0.0–10.0)	
n	2,243	
Uterine contraction		
Mean±SD	1.2±1.3	
Median (minimum–maximum)	1.0 (0.0–8.0)	
n	1,531	
Type of labor	976	100
Spontaneous	759	77.8%
Induced, successful	114	11.7%
Induced, unsuccessful	103	10.5%
A healthcare professional during labor	976	100
Only physician	300	30.7%
Only nurse	80	8.2%
Physician and nurse	430	44.1%
Without information	166	17.0%
Type of delivery	2,435	100
Vaginal	570	23.4%
Cesarean section	1,865	76.6%
Healthcare professional during delivery	2,435	100
Physician	2,417	99.3%
Nurse	18	0.7%
Healthcare professional during labor and delivery	2,435	100
The same professional of prenatal care	1,305	53.6%
One professional of the prenatal care team	85	2.5%
One professional of the hospital emergency care team	1,034	42.5%
Other	11	0.5%

SD: standard deviation.

Regarding decision-making, 86.5% of women reported that the mode of delivery was decided by themselves alone or jointly with the healthcare team, based on self-reported information obtained during postpartum interviews. Among cesarean sections, 23.3% were reportedly decided during prenatal care, 9.1% at hospital admission, and 61.4% during labor or in the delivery room.

## DISCUSSION

The World Health Organization (WHO)’s designation of 2020 as the “Year of the Nurse and the Midwife” emphasized the importance of these professionals in maternal and child health^10^. Our findings show a limited participation of nurse-midwives in private maternity hospitals participating in the Adequate Childbirth Project. Fewer than one-third of vaginal deliveries were attended by nurse-midwives, a proportion lower than that reported in public maternities linked to the Rede Cegonha^11^ and in the “Birth in Brazil” study^12^.

Brazilian law authorizes nurse-midwives to independently manage low-risk births (Law No. 7,498/1986; Decree No. 94,406/1987), and their role in providing respectful, humanized, and evidence-based care is well established^13,14,15^. International studies consistently associate nurse-midwife-led care with fewer interventions, improved maternal and neonatal outcomes, and reduced mortality, particularly in low- and middle-income countries^8,16^. Accordingly, the WHO and Brazil’s Ministry of Health recommend integrating nurse-midwives into labor and delivery care^10,17,18,19^. However, the present study design does not allow establishing causal relationships between professional participation and delivery outcomes.

In this study, participants were predominantly white, well educated, and of higher socioeconomic status, reflecting the private healthcare context and limiting generalizability. Although many women initially preferred vaginal delivery, cesarean section rates remained high. Most women were not in labor at admission, nearly all births were physician-attended, and continuity of prenatal and intrapartum care was limited—patterns similar to those reported nationally^5^. Although nurse-midwives were present during labor in about half of cases, they rarely assisted delivery, indicating limited integration despite their formal presence^12,20^.

Cesarean decisions were frequently made during prenatal care or jointly with physicians, consistent with high elective cesarean rates in private hospitals reported in national studies^21^. The discrepancy between initial preference for vaginal delivery and the observed outcomes may reflect institutional routines, provider practices, and organizational factors within private care settings. In this context, models of care in the private sector—often centered on physician-led continuity and, in some cases, privately hired teams—may influence the degree of involvement of obstetric nurses. Their participation may vary depending on whether they are employed by hospitals, affiliated with health insurance providers, or independently contracted by patients. The WHO has warned that even low-risk women may be exposed to iatrogenic interventions, particularly in medicalized care models, where resistance to nurse-midwife integration may persist^22^.

Regardless of socioeconomic status or low obstetric risk, women may be exposed to iatrogenic risks during childbirth. This was emphasized in the WHO’s 2015 statement on cesarean section rates^23^, which noted that women from higher socioeconomic groups may be more prone to medicalized care and unnecessary interventions. In such contexts, nurse-midwives may face barriers to full integration into the childbirth care model^22^.

Globally, shortages of nurse-midwives remain substantial, prompting the WHO’s Global Strategic Directions for Nursing and Midwifery 2021–2025 to strengthen education, employment, leadership, and service delivery^6,24^. In Brazil, the Rede Cegonha reinforced the legal and clinical role of nurse-midwives in low-risk childbirth. However, this study has limitations: it was restricted to six private hospitals, predominantly in São Paulo, and based on secondary data. Its descriptive design does not allow assessment of associations or control for confounding factors. Despite these limitations, the findings provide relevant insights into the organization of childbirth care in the Brazilian supplementary health system.

## CONCLUSION

Nurse-midwives remain underutilized in private maternity hospitals, despite strong evidence supporting their role in evidence-based, humanized childbirth care. Their limited participation observed in this study highlights a gap between recommended care models and current practice in the private sector. Although causal relationships cannot be established, expanding nurse-midwife involvement in low-risk births, supported by health policies and institutional protocols, may contribute to improving care quality, promoting appropriate use of interventions, and advancing woman-centered maternal and neonatal health outcomes.

## Data Availability

The datasets generated and/or analyzed during the current study are available from the corresponding author upon reasonable request.
